# Rehabilitation Strategies for Wernicke-Korsakoff Syndrome: Physiotherapy Interventions and Management Approaches

**DOI:** 10.7759/cureus.53948

**Published:** 2024-02-10

**Authors:** Ghanishtha C Burile, Neha Arya, Nikita H Seth, Tejaswini Fating

**Affiliations:** 1 Community Health Physiotherapy, Ravi Nair Physiotherapy College, Datta Meghe Institute of Higher Education and Research, Wardha, IND

**Keywords:** wernicke-korsakoff syndrome, thiamine deficiency, cognitive rehabilitation, gait training, electrical stimulation sensorimotor training, frenkel's exercise, brain gym exercises, benson relaxation technique

## Abstract

Korsakoff syndrome and Wernicke's encephalopathy (WE) show neurological and cognitive deficits. Wernicke-Korsakoff syndrome (WKS) is a compound neurological condition. The cause of this neurological condition could be the consumption of alcohol regularly for a chronic duration. A tailored rehabilitation protocol that focuses on cognitive and physical deficiencies was implemented along with thiamine supplementation for managing a case of a 49-year-old male patient who had a history of high alcohol consumption and was exhibiting typical signs of WKS. After planning a proper physiotherapy plan, it is necessary to look after the patient's progress along with re-evaluation, which reveals notable gains in cognitive function, memory, and functional independence. There is a dearth of research on the impact of physical therapy in managing WKS. The above case report reflects the benefits of combining physiotherapy, cognitive rehabilitation, and balance training to improve patient functionality and independence. Tailored rehabilitation interventions like the Benson relaxation method (BRM), brain gym exercises, Frenkel's exercise, electrical stimulation, sensorimotor training, basic body awareness therapy (BBAT), and gait training can be used to enhance a patient's quality of life. Addressing individual needs is essential in managing WKS, focusing on the importance of comprehensive care beyond cognitive rehabilitation alone.

## Introduction

Wernicke-Korsakoff syndrome (WKS) is a condition in which there is neurological as well as cognitive dysfunction due to thiamine deficiency. Korsakoff syndrome is characterized by a chronic thiamine deficiency that primarily affects memory development [1]. Thiamine deficiency can lead to symptoms like a sudden increase in heart rate with palpitations, decreased diet, dizziness, fatigue, irritability, weight loss, and bladder retention. The traditional triad of Wernicke's encephalopathy, which includes confusion or delirium and anticholinergic autonomic dysfunction, can be responsible for these symptoms [2]. An autopsy-based series reveals that about 82% of patients experience changes in their mental condition, and about 23% of patients experience incoordination of gait and truncal ataxia, which are caused by vestibular dysfunction and cerebellar vermis involvement [3]. Growing research on this topic suggests that astrocytes have a role in the pathophysiology of thiamine deficiency. The excitotoxic event is discussed in terms of the gamma-aminobutyric acid (GABA) transporter subtype GABA transporter 3 (GAT-3), changes in other astrocytic proteins, such as glial fibrillary acidic protein (GFAP) and glutamine synthetase, and changes in glutamate uptake and levels of the astrocytic glutamate transporters excitatory amino acid transporters (EAATs) 1 and 2 in thiamine deficiency and Wernicke's encephalopathy [4]. The patients who are alcohol dependent have lower thiamine levels. The processes are assumed to be poor food, along with alcohol's effects on thiamine absorption, storage, activation, and excretion [5].

When managing patients with WKS, individual-specific physical rehabilitation measures should be taken into account in addition to cognitive rehabilitation [6]. Basic body awareness therapy (BBAT) is a useful physiotherapy technique for treating balance issues, cerebellar issues, and the incoordination of the gait associated with WKS. The BBAT approach might offer a fresh viewpoint for the rehabilitation of WKS hunger strike survivors [7]. The most frequently afflicted cranial nerve is palsy of the sixth nerve. This results in diplopia and the deconjugate gaze [8]. When creating a long-term treatment plan for individuals in the rehabilitation phase or receiving domiciliary management, it is imperative to consider the cognitive aspects of chronic alcoholics and address their challenges with everyday living activities [9]. Individuals suffering from an alcohol-related brain injury can have good results from the services of community teams and centres who experience similar symptoms of alcohol withdrawal and suffer from cognitive and neurological impairment. Alcohol harms the brain. An organized rehabilitation procedure serves as a foundation for handling the aforementioned issues [10].

## Case presentation

Patient information

The case involves a 49-year-old male presenting with symptoms of reduced appetite, insomnia, excessive daytime sleepiness, altered sensorium, visual hallucinations, generalized weakness, and loss of balance and coordination. The patient had a significant 15-year history of high alcohol consumption over the past two to three years. The patient's current complaints were similar to a previous episode characterized by unsteadiness, poor concentration, agitation, dysarthria, memory disorder, and ataxic gait. So the patient was brought to the psychiatric department where investigations like ultrasonography of the abdomen and pelvis and vitamin B1 levels were monitored. It was found that the thiamine levels were less than normal (normal thiamine levels in a healthy individual are 70-180 nmol/L). The patient was diagnosed with WKS. Neurological assessment, including reflexes, tone, and cognitive functions, was done. The findings prompted the patient's transfer to the neurology ICU, where a treatment regimen was initiated. Thiamine was administered intramuscularly at 500 mg three times daily for one week, followed by an oral maintenance dose of 250 mg/day. Additionally, the patient was referred to the physiotherapy department for comprehensive care.

Clinical findings

A neurological examination was performed, which included an assessment of muscle tone and was found to be normal. Evaluation of non-equilibrium coordination tests, equilibrium coordination tests, and reflexes was assessed. The assessment of non-equilibrium coordination tests has been presented in Table 1.

**Table 1:** Assessment of non-equilibrium coordination tests 1 (severe impairment): Able only to initiate activity without completion; movements are slow with significant unsteadiness, oscillations, and extraneous movement. 3 (minimal impairment): Able to accomplish an activity, with slightly less than normal control, speed, and steadiness.

Non-equilibrium coordination test	Pre-operative	Post-operative
Finger to nose	1	3
Finger to finger	1	3
Rebound phenomenon	1	3

The assessment of the equilibrium coordination test has been presented in Table 2.

**Table 2:** Assessment of equilibrium coordination test Poor - for static balance: Requires handhold support and moderate to maximal assistance to maintain position. For dynamic balance: Unable to accept the challenge or move without loss of balance. Good - for static balance: Able to maintain balance without handhold support, limited postural sway. For dynamic balance: Accepts moderate challenge, able to maintain balance while picking objects off the floor.

Equilibrium coordination test	Pre-operative	Post-operative
Tandem walk	Poor	Good
March on place	Poor	Good
Sitting on a therapy ball	Poor	Good

The assessment of reflexes has been presented in Table 3.

**Table 3:** Reflex assessment 3+ indicates hyperactive.

Reflexes	Right	Left
Biceps reflex	3+	3+
Triceps reflex	3+	3+
Supinator reflex	3+	3+
Knee reflex	3+	3+
Achilles reflex	3+	3+
Plantar reflex	3+	3+

Physiotherapy rehabilitation protocol

The patient was given physical rehabilitation for one hour per day for four weeks. Table 4 depicts a summary of the intervention protocol that was followed throughout the rehabilitation programme.

**Table 4:** Structured physiotherapy protocol ROM: range of motion; BRM: Benson relaxation method; BBAT: basic body awareness therapy. References [11-18].

S. No.	Problem list	Goal	Interventions
1	Lack of knowledge about the condition	Patient education and awareness	The patient was made aware of his condition and the importance of physiotherapy was told to the patient and their relatives to improve the patient’s quality of life
2	Cognitive impairments like learning and memory	To improve cognitive functioning	Brain gym exercises include the following: hookups - this method helps in mind and body relaxation (for two minutes, five sets of eight repetitions); for improving eye muscle control, balance, and concentration, lazy eight exercises can be given; Earth buttons aids in mental alertness and whole-body alignment (10 repetitions for two minutes)
3	Lack of postural stability	To improve static and dynamic postural stability	Sensorimotor training for correcting muscular imbalance includes wall slides and core exercises. Each session was conducted for 10 minutes of warm-up exercises (50-60 minutes of the exercise period) and 5-10 minutes of cool down. Cool-down exercises include deep breathing exercises, abdominal breathing, and mild stretching
4	Loss of balance	To improve balance and coordination	Frenkel’s exercise in standing: Walking forward, walking sideways and returning to the original position, walking backwards, and a combination of dual-task, function-oriented challenges while controlling balance
5	Gait altered	To improve coordination	Gait training that includes normal walking on foot marks tandem walking, walking across obstacles, walking over obstacles, sideway walking
6	Weakness in knee and foot extensor muscles	To improve knee and foot extensor strength	Electrical stimulation for strengthening knee and foot extensor muscles
7	Reduced aerobic capacity	To improve aerobic capacity	Walking, when opposed to treadmill walking, cycle ergometer requires less postural control and may be a better option for those with weak balance, aerobic and strengthening exercises are combined in a training routine to promote aerobic capacity
8	Reduced functional mobility	To improve functional mobility	Activities like getting up from a chair, climbing stairs, or reaching for objects
9	Reduced range of motion	To improve range of motion	ROM exercises
10	Anxiety	To reduce symptoms of anxiety	In the Benson relaxation method (BRM), the patient should be in a comfortable sitting position, closing the eyes, deeply relaxing all the muscles, starting from the feet and progressing up to the face, breathing through the nose while becoming aware of one's own breath (10 minutes in the morning and evening to gain the health benefits associated with relaxation), touch-based skills reduce levels of anxiety and depression, breathing exercises would help to reduce the mean value of fatigue
11	Alterations in body awareness	To cope with pain anxiety. For good balance and stability	Basic body awareness therapy (BBAT) for increasing movement awareness progressing towards less effort and a better function. The therapy programme includes movements from everyday life that include lying, sitting, standing, and walking for improving activities of daily living to gain more functional movement quality and improve posture
12	Apathy	To reduce the symptoms of apathy	Patients may find that music helps them express their feelings, diverts their attention from bad moods, and teaches them new cognitive, psychosocial, and physical skills
13	Patients with difficulty in learning, repetitive errors while performing activities	Promotes learning without errors	Errorless learning strategy

**Figure 1:**
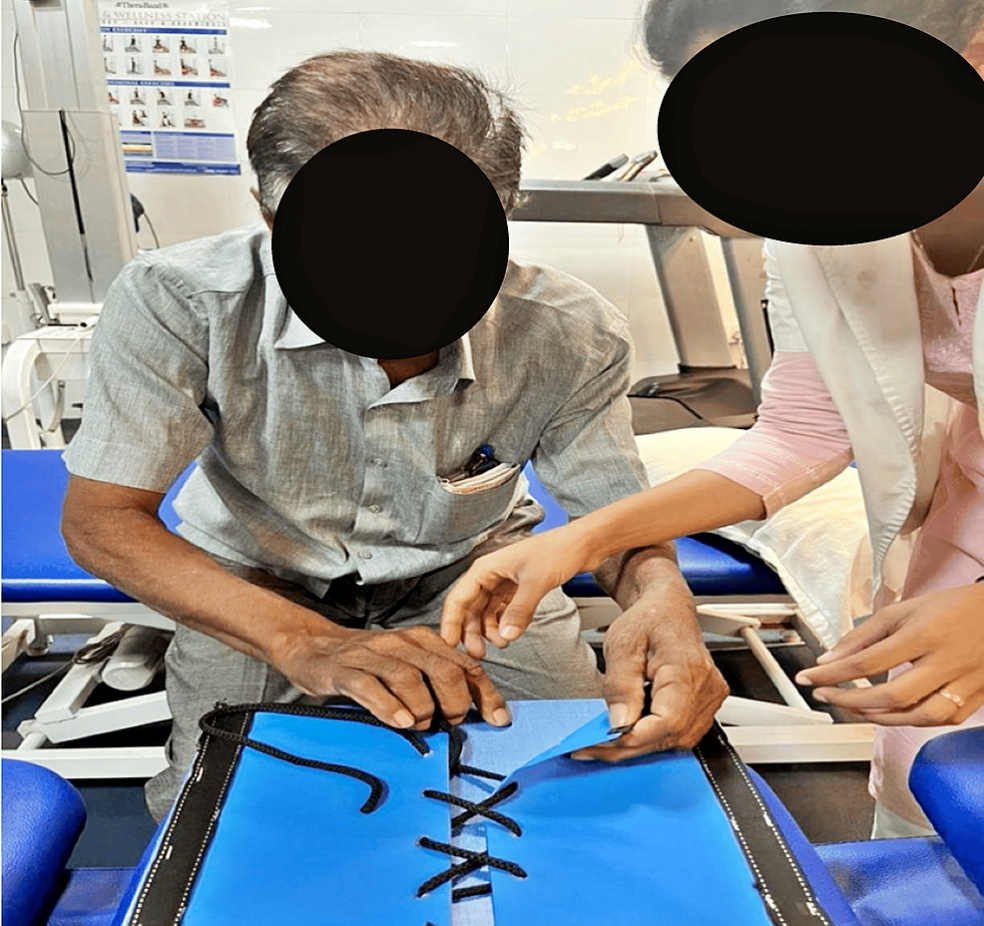
The patient was assisted while performing fine motor movements

Figure 1 depicts the patient being assisted while performing fine motor movements to improve motor functions.

**Figure 2:**
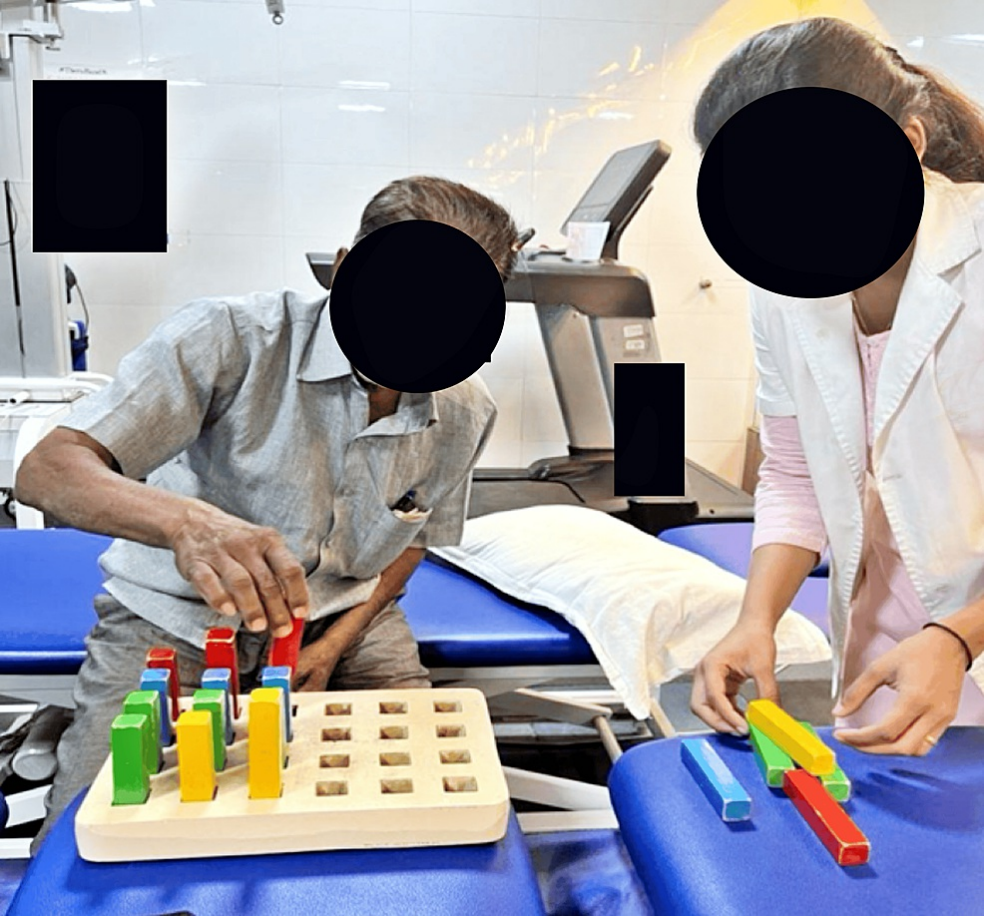
The patient performing fine motor movements on peg board

Figure 2 depicts the patient performing fine motor movements on a peg board.

**Figure 3:**
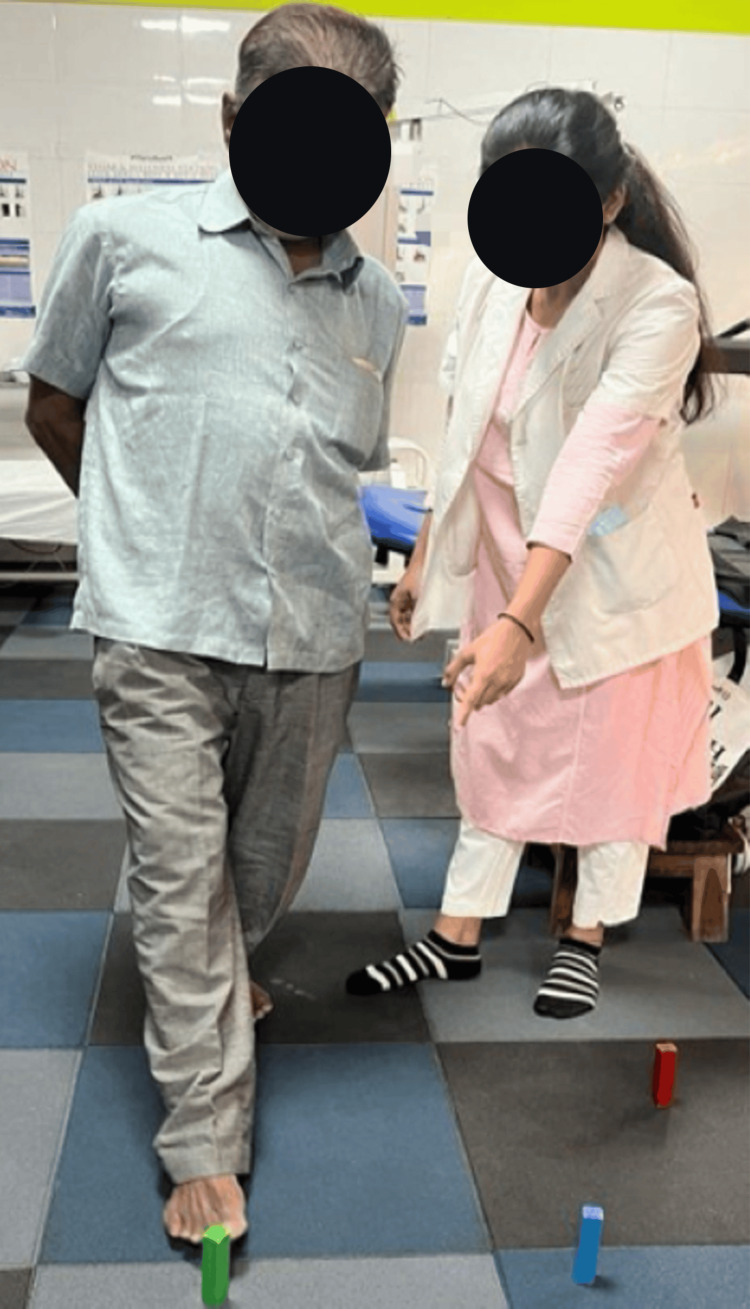
Therapist assisting the patient in performing Frenkel's exercise for lower limb

Figure 3 depicts the therapist assisting the patient in performing Frenkel’s exercise in standing and reaching for the objects that are pointed by the therapist's fingers. Frenkel's exercises are a series of gradual progressive exercises designed to increase coordination and used to retrain proprioception, particularly focused on the lower limbs.

**Figure 4:**
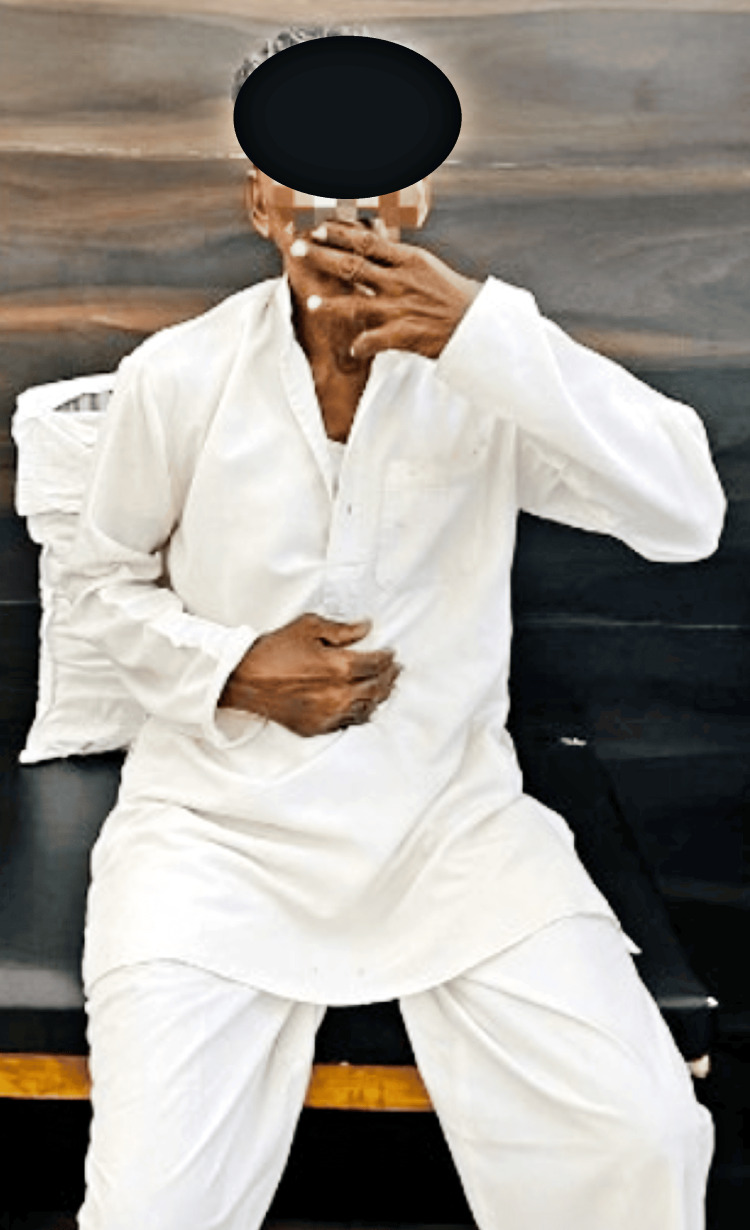
The patient demonstrating the Earth buttons exercise that helps in improving mental alertness and whole-body orientation

Figure 4 depicts the patient demonstrating the Earth buttons exercise that helps in improving mental alertness and whole-body orientation.

Follow-up and outcome measures

In the case report, significant improvement was observed after treatment across various outcome measures. Cognitive function was assessed by the Montreal Cognitive Assessment (MoCA), while the Rivermead Behavioural Memory Test (RBMT) was used to assess behavioural changes and memory functioning, and manual muscle testing was used to determine the strength of muscles. Additionally, functional independence, evaluated through the Functional Independence Measure (FIM), indicated positive treatment outcomes after rehabilitation as compared to before, as mentioned in Table 5.

**Table 5:** Outcome measures MoCA: Montreal Cognitive Assessment; RBMT: Rivermead Behavioural Memory Test; FIM: Functional Independence Measure.

Outcome measures	Before treatment	After treatment
MoCA	9/30	20/30
RBMT	0/24	6/24
FIM	Maximal dependence	Modified dependence

Muscle strength assessment was done according to the modified Oxford Grading System, as depicted in Table 6.

**Table 6:** Manual muscle testing 2+: initiates motion against gravity; 3: complete range of motion against gravity.

Movements of muscles of the joint	Pre-intervention	Post-intervention
Shoulder flexors	2+	3
Wrist flexors	2+	3
Hip flexors	2+	3
Hip extensors	2+	3
Knee flexors	2+	3
Knee extensors	2+	3
Ankle flexors	2+	3
Ankle extensors	2+	3

## Discussion

The case report reveals the complex neurological condition of WKS, often stemming from chronic alcohol consumption, and the multifaceted rehabilitation strategy implemented for a 49-year-old male patient exhibiting typical signs of the syndrome. Therapy for WKS has not received much attention. WKS is treated with cognitive rehabilitation and thiamine replacement therapy, which has no set dosage and can vary depending on patients' symptoms. Physiotherapy can be used as a primary goal for managing primary treatment strategy in subjects with vascular dementia and dementia with Lewy bodies is needed to demonstrate the efficacy among these populations with the help of gait, balance, and cognitive therapy [19]. Combining dual-task and function-oriented challenges while controlling balance stimulates the sensory and neuromuscular control mechanisms. In some subjects, these programmes have been found to improve static and dynamic stability, as well as several aspects of the quality of life. They have recently been found to improve cognitive functions such as memory and spatial cognition [20].

The above case outlines the cognitive and physical deficits it presents and highlights the scarcity of research regarding the impact of physical therapy in managing this syndrome. Investigations like ultrasonography and vitamin B1 levels contribute to the diagnosis. Outcome measures assessed through cognitive assessments, memory tests, and functional independence scales show significant improvement post-treatment, underlining the effectiveness of the implemented rehabilitation protocol. The core of the case report focuses on the physiotherapy rehabilitation protocol tailored to the patient's specific deficits. The details of a comprehensive plan spanning cognitive exercises, sensorimotor training, gait and balance exercises, muscle strengthening via electrical stimulation, aerobic conditioning, range of motion exercises, and methods to address emotional aspects such as anxiety, alterations in body awareness, and apathy are presented. Visual aids, including images of therapeutic interventions like Frenkel's exercise, balance training, and fine and gross motor movements, enrich the case report's depth, illustrating the practical application of these interventions. Conclusively, the case reports the significant holistic rehabilitation approach for WKS, stressing the synergy between cognitive rehabilitation, physical therapy, and emotional support in enhancing patient functionality and independence. It highlights the necessity of individualized care plans and the potential role of physiotherapy in improving the quality of life for individuals afflicted by this complex neurological condition.

## Conclusions

Integration of cognitive and physical functions in post-acute care is necessary to achieve maximum independence. To reduce recurring symptoms post-operatively, a good physical therapy programme, which mainly focuses on cognitive impairment, gait, and balance, is required. Strengthening rehabilitation programme, along with monitoring neuropsychological assessments before and after rehabilitation, will improve the patient's confidence in performing activities of daily living, which is necessary in Korsakoff syndrome. This case emphasizes on early and focused physical therapy rehabilitation programmes that can help patients to recover neurologically and regain optimal motor function, which in turn improves their general health and quality of life.
